# Predictive factors for successful clinical outcome 1 year after an intensive combined physical and psychological programme for chronic low back pain

**DOI:** 10.1007/s00586-013-2844-z

**Published:** 2013-06-16

**Authors:** Miranda L. van Hooff, Maarten Spruit, John K. O’Dowd, Wim van Lankveld, Jeremy C. T. Fairbank, Jacques van Limbeek

**Affiliations:** 1 Sint Maartenskliniek, P.O. Box 9011, 6500 GM Nijmegen, The Netherlands; 2 The RealHealth Institute, 23-31 Beavor Lane, London, W6 9AR UK; 3 HAN University of Applied Sciences, P.O. Box 6960, 6503 GL Nijmegen, The Netherlands; 4 Nuffield Department of Orthopaedics, Rheumatology and Musculoskeletal Sciences, Nuffield Orthopaedic Centre, University of Oxford, Windmill Road, Oxford, OX3 7HE UK

**Keywords:** Low back pain, Disability, Prediction, Pain management, Cohort study, Outcome

## Abstract

**Purpose:**

The aim of this longitudinal study is to determine the factors which predict a successful 1-year outcome from an intensive combined physical and psychological (CPP) programme in chronic low back pain (CLBP) patients.

**Methods:**

A prospective cohort of 524 selected consecutive CLBP patients was followed. Potential predictive factors included demographic characteristics, disability, pain and cognitive behavioural factors as measured at pre-treatment assessment. The primary outcome measure was the oswestry disability index (ODI). A successful 1-year follow-up outcome was defined as a functional status equivalent to ‘normal’ and healthy populations (ODI ≤22). The 2-week residential programme fulfills the recommendations in international guidelines. For statistical analysis we divided the database into two equal samples. A random sample was used to develop a prediction model with multivariate logistic regression. The remaining cases were used to validate this model.

**Results:**

The final predictive model suggested being ‘in employment’ at pre-treatment [OR 3.61 (95 % CI 1.80–7.26)] and an initial ‘disability score’ [OR 0.94 (95 % CI 0.92–0.97)] as significant predictive factors for a successful 1-year outcome (*R* ^2^ = 22 %; 67 % correctly classified). There was no predictive value from measures of psychological distress.

**Conclusion:**

CLBP patients who are in work and mild to moderately disabled at the start of a CPP programme are most likely to benefit from it and to have a successful treatment outcome. In these patients, the disability score falls to values seen in healthy populations. This small set of factors is easily identified, allowing selection for programme entry and triage to alternative treatment regimes.

## Introduction

Chronic low back pain (CLBP) is a major cause of distress and disability and in the Netherlands CLBP accounts for considerable healthcare and socioeconomic costs [1, 2]. CLBP is defined as back symptoms persisting for at least 3 months [3] and these symptoms are associated with persistent or recurrent disability. Multiple studies have emphasised the psychosocial influence on the development of chronicity and the persistence of pain complaints [4–6]. Increased distress accompanies more severe pain, enhances pain-related disability and contributes to the development of chronicity of LBP [7–9]. Some evidence suggests that fear of movement [8] and catastrophizing [8, 10, 11] play a role when pain has become persistent.

In line with these findings, international guidelines [12–14] and a Cochrane review [15] have recommended multidimensional interventions using a cognitive behavioural approach to improve psychological and physical functioning. However, most of the interventions studied show only small, short-lived effects [7, 15–17]. One explanation for these small effects could be the heterogeneity of the CLBP population studied. Although the aetiology of chronic low back pain remains unknown, it has been suggested that several subgroups could be identified amongst CLBP patients who are likely to benefit from specific recommended interventions [14]. It is possible that the efficacy of the interventions employing physical and cognitive behavioural approaches would be improved by matching interventions to patient characteristics.

Multiple studies, including several systematic reviews, have studied patient characteristics to identify potentially predictive factors for treatment outcome in CLBP [6, 18, 19]. In the most recent review, van der Hulst et al. [6] analyzed the prognostic value of numerous biomedical, demographic and psychosocial factors in 17 internally valid studies (*n* = 3,356) to determine the multidisciplinary rehabilitation treatment outcome in patients with non-specific CLBP. Due to methodological flaws in the included studies, they were not able to define a generic set of predictive factors. These methodological problems include heterogeneity in the study populations, the high number of prognostic factors, and the wide variety of treatment and outcome measures. Against this background, more research related to the subject is warranted.

A recent study reviewed the results of a short, intensive, two-week residential combined physical and psychological (CPP) programme for patients with longstanding CLBP who were not eligible for spinal surgery. The main goal of that programme is to improve daily functioning. On average, there was a large, clinically relevant improvement in terms of both disability and quality of life. Both remained stable during the following 12 months [20]. Two years after participation in that programme, not only did these post-treatment improvements remain consistent, there was a substantial reduction in healthcare use and an increased return to work [21]. Although large effect sizes for disability had been found, the study showed a wide range in the improvements of disability among participants. Identifying the predictive factors associated with improvement in disability would enable the selection of those CLBP patients who are most likely to benefit from such a programme, and ultimately, to develop treatment regimes for those who will not.

The purpose of this longitudinal study is to determine those factors pre-treatment that predict a successful CPP programme outcome. We defined successful outcome as a clinically relevant and consistent improvement at 1-year follow-up towards the values seen in healthy populations. The data from 524 consecutive CLBP patients were used to answer this. We expected that the pre-treatment degree of experienced pain intensity, belief in the ability to manage and to cope with CLBP complaints, the degree of disability, and employment status were the most likely predictive factors for treatment outcome. We expected high psychological distress to be an indicator of poor outcome [8, 22, 23].

## Methods

### Study design

The predictive value of patient characteristics, including disability, pain severity, cognitive and behavioural factors were analysed prospectively. These analyses were related to the patient’s disability 12 months following the 2-week residential CPP programme.

### Patients

The study group consisted of patients with CLBP referred to a tertiary orthopaedic hospital, specialised in spine care. Patients who had not improved following conservative treatment delivered in primary care [21] and who were not eligible for spinal surgery or invasive pain management were referred by the spine surgeons for the CPP programme. The main inclusion criteria were (1) low back pain for at least 6 months, (2) age between 18 and 65 years, (3) willingness to change behaviour, (4) willingness to follow the 2-week programme in a hotel facility, and (5) able to speak and read Dutch. The main exclusion criteria were involvement in litigation and/or compensation claims and psychiatric disorders as formally and primarily diagnosed by psychiatrists, in accordance with the DSMIV classification. Final inclusion was based on an extensive intake procedure and all patients were assessed by a multidisciplinary team consisting of a psychologist, a physiotherapist, an occupational therapist, and a movement teacher.

### Intervention

The CPP programme is NICE guidelines compliant [14]; it is a residential 2-week programme including a cognitive behavioural approach. The programme runs in collaboration with the spine surgeons. The group-orientated training sessions in the 2-week programme are delivered by a multidisciplinary team, extensively trained in cognitive behavioural techniques for chronic pain. The programme involves 100 h of patient contact time, delivered in a group-orientated residential setting, including 40 h of cognitive behavioural training, 30 h of physical activities, and a 10 h of education. It includes a pre-treatment assessment day, the 10-day residential programme, and 1 day follow-up assessments at 1 and 12 months post-treatment. The main goal of the intervention is to improve daily function, and this is made clear to the patient. This goal is achieved by increasing the participant’s ability to self-manage, addressing the psychological impact of pain, and increasing physical condition; all are directed towards decreasing disability and thus enhancing future return to work. A more detailed description of the intervention is reported in a previously published article [20].

### Outcome measures

We used a self-report questionnaire at pre-treatment, at the end of the 2-week residential programme (post-treatment), and at 12-months follow-up. These assessments are an integral part of the programme. At pre-treatment assessment, participants provided information on medical history, pain history, pain scores, consumption of pain medication, and employment status.

In this study, age was categorised in tertiles (years; age ≤42, 43–50, >50). We dichotomised values for the consumption of pain medication and employment status (‘employed’) into (1 = yes; 0 = no). At each assessment, participants completed questionnaires on functional status, pain severity, psychological distress, self-efficacy, pain catastrophizing, and fear of movement. All these self-report measures have previously been validated in CLBP samples.

#### Self-report measures

The primary outcome variable for this study is functional status as measured by the oswestry disability index (ODI, version 1.0 in Dutch) [24]. The ODI measures the impact of LBP on daily functioning in ten domains of daily life. In ‘normal’ healthy populations, the weighted mean ODI score is 10 (SD range 2–12) and in chronic back pain 43.3 (SD range 10–21) [25].

We used several secondary outcome variables to quantify aspects of physical and psychosocial functioning.

1. Current pain severity was assessed with the Numeric Rating Scale (NRS_severity_) [26], which is used to measure the experienced intensity of pain. The ordinal scores range from 0 to 100, with higher scores indicating higher levels of pain intensity.
2. The modified Zung Self-Rating Depression Scale (ZSDS) [27] is an indicator of psychological distress and depression. Patients are asked to rate 23 items on a four-point ordinal scale. Total scores range between 0 and 69, with higher scores indicating higher levels of depressed mood. For patients with CLBP, the following classification has been given: <17 ‘normal’, 17–33 ‘at risk’ and >33 ‘depressed mood’ [28].
3. We used the pain self-efficacy questionnaire (PSEQ, Dutch translation) [29] to measure the strength of the patient’s belief about the ability to accomplish a range of activities despite the pain. The PSEQ is a ten-item inventory with responses rated on a seven-point ordinal scale. The total score ranges from 0 to 60, with higher scores indicating higher perceived self-efficacy beliefs.
4. Dysfunctional cognitive behavioural factors (i.e. pain-related catastrophizing and fear of movement) were assessed with the Pain Catastrophizing Scale (PCS) and the Tampa Scale for Kinesiophobia (TSK). We used a Dutch translation of the PCS based on the original version [30]. The items are scored on a five-point ordinal scale. The total score is between 0 and 52 points, with higher scores reflecting higher levels of pain catastrophizing. Fear of movement/(re)injury in individuals with pain was measured by the TSK [27, 31, 32]. The unweighted sum score ranges between 17 and 68 points, with higher scores indicating higher levels of fear of movement.

### Data analysis

#### Descriptive analysis

Pre-treatment patient characteristics were descriptively summarised, with categorical data in count and percentages and continuous variables as means and standard deviations. The percentage non-responders were calculated and the pre-treatment data of non-responders and responders compared. To evaluate differences between both groups at pre-treatment, we used Chi square tests for categorical variables and independent Student’s *t* tests for continuous variables.

#### Definition of ‘successful treatment outcome’

The ODI as the primary outcome measure was used to define successful treatment. ‘Normal’ healthy populations have an ODI mean score of 10 (SD 2–12) [25]. Therefore, being successful was defined as having reached a maximum of 22 points on the ODI at the 1-year follow-up, including the maximum reported standard deviation of 12 points (mean plus 2 SD). The scores of the patients were then dichotomised into ‘success’ (value = 1) and ‘failure’ (value = 0) for the programme, and both groups were compared to pre-treatment characteristics.

#### Prediction analysis

We considered all pre-treatment variables as factors that could influence the outcome. These influencing factors were identified by a prediction model. To develop the prediction model, associated factors were first identified using Pearson’s correlation coefficients. To predict those factors with contribution to the probability of a successful outcome, a univariate logistic regression analysis was performed. Subsequently, we randomly divided the complete dataset into two equal samples. One sample was used to develop the final prediction model, and the second was used to validate that model. The final prediction model with odds ratios (OR) and 95 % confidence intervals (95 % CI) for predictive factors was based on a multivariate logistic regression analysis. The dichotomised primary outcome variable disability (success/failure) was used as the dependent variable in this model. In one block, the identified and significant pre-treatment patient characteristics and pre-treatment values on secondary outcomes were entered into the model as independent variables. A forward, stepwise selection method was used for analysis. The procedure starts with the independent variable that correlates most strongly with the dependent variable. Subsequently, the next independent variable is selected and added to the final model. The remaining cases were used to validate the final prediction model that had been developed. For this procedure, the identified predictive factors from the model developed were entered into the model as independent variables.

This final prediction model was then used to calculate a pre-treatment probability as to whether an individual patient belongs to the group that will have a successful treatment outcome. This is estimated with the formula: *p* (success/failure) = e^*f*(*x*)^/1 + e^*f*(*x*)^. For this purpose, all identified significant predictor variables were included in the logistic function: *f*(*x*) = *a* + *b*_1_ *x*_1_ + *b*_2_ *x*_2_ … *b*_k_ *x*_k_. The calculated probabilities are between 0 and 1, and interpreted as follows: <0.5 probability in favour of failure, >0.5 probability in favour of success, and 0.5 equal likelihood for either outcome.

In the literature, it has been hypothesised that patients with high psychological distress have a poor outcome [22], and more specifically, that the level of depressed mood contributes to pain-related disability [7, 8]. Where in the above multivariate logistic regression analysis, psychological distress appeared to be a predictive value for treatment outcome, additional separate analyses are performed.

All data analysis was performed using SPSS version 18.0. An *α* of 0.05 was considered statistically significant. A scatter plot to give an illustration of disability in the study sample was created in STATA version 10.0.

#### Missing data

As self-report questionnaires were used as outcome measures, we expected missing data during the follow ups. To handle such missing data, the multiple imputation (MI) method was used under the assumption that the data were ‘missing at random’. This implies that the missing data are related to other observed or documented patient data but not to unobserved outcomes. The MI-technique replaces each missing value of the incomplete data set with a set of plausible values (*n* = 10, current study), derived from the available data. These values represent the uncertainty in the correct value to impute. To generate these values to impute, the data augmentation Markov chain Monte Carlo replacement method was used. In this study, ten datasets were generated; each generated dataset was analysed according to the previously mentioned statistical tests. Overall means, beta weights and standard errors were calculated.

## Results

### Study population

**Fig. 1:**
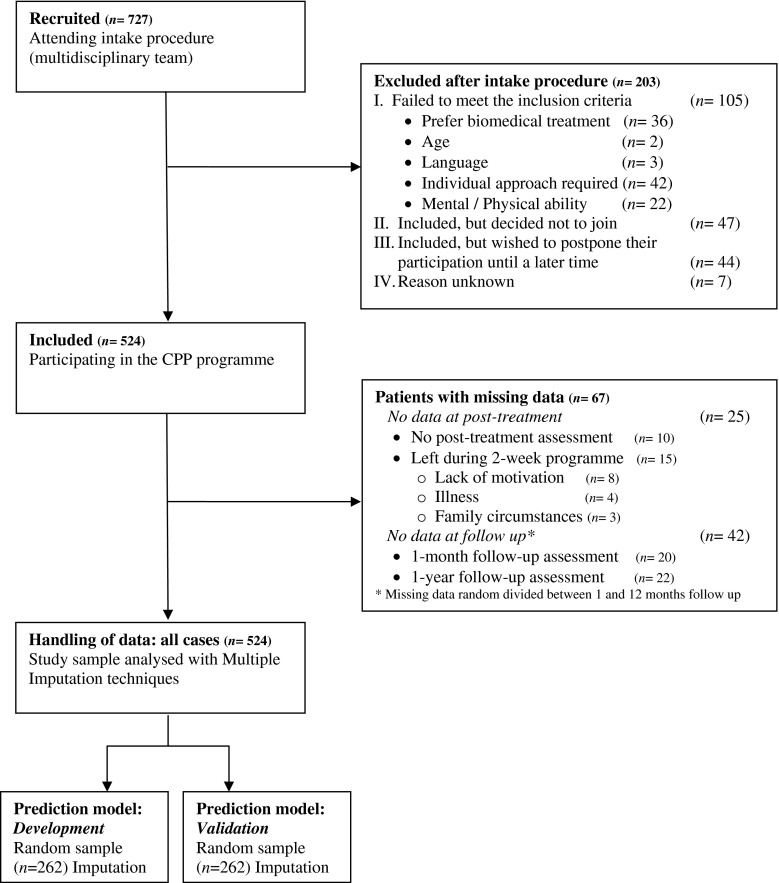
Flow diagram of CLBP patients recruited by the spine surgeons at the outpatient orthopaedic department for the CPP programme and handling of the data of these patients

The spine surgeons recruited 727 CLBP patients for pre-treatment assessment. Between October 2006 and January 2011, 524 patients (72.1 %) were included and participated in the programme. Of this sample (*n* = 524), 67 patients (12.8 %) had data missing from at least one assessment after the pre-treatment assessment. The flow diagram (Fig. 1) shows the available patient data at each stage of the study. At post-treatment assessment, data for 25 patients were missing. These included the first group of ten patients where this assessment had not been conducted. In addition, 15 patients left during the 2-week residential programme. The missing data of the remaining 42 patients were randomly divided between the 1- and 12-month assessments. These 67 (25 + 42) patients with missing data are not significantly different from the patients with complete data sets with regard to pre-treatment characteristics and the pre-treatment scores on the various outcome measures: ODI, PCS, TSK, NRS_severity_, ZSDS, and PSEQ.

General pre-treatment characteristics for the complete study population (*n* = 524) are given in Table 1. The reported mean age was 45 (±9.6) years; a small majority was female (58 %). The mean LBP duration was 13 (±10.8) years, indicating that our study population had longstanding CLBP. At pre-treatment assessment, two-thirds of the patients were at work (68 %); one-third had undergone surgery for LBP.

**Table 1:** Pre-treatment characteristics reported by patients with CLBP participating in the CPP programme (*n* = 524)

Pre-treatment characteristics categorical variables	Total (*n* = 524) *n* (%)	Disability	
		Success^a^ (*n* = 217) *n* (%)	Failure^a^ (*n* = 307) *n* (%)
Sociodemographic			
Gender, female	303 (57.8)	118 (54.4)^b^	185 (60.3)^b^
Employment status, yes	356 (67.9)	194 (89.4)^c^	162 (52.8)^c^
CLBP history			
Pain medication, yes	454 (86.6)	176 (81.1)^d^	278 (90.6)^d^
Previous surgery, yes	169 (32.3)	54 (24.9)^e^	115 (37.5)^e^

Pre-treatment characteristics continuous variables	Total (*n* = 524) mean (SD)	Disability	
		Success^a^ (*n* = 217) mean (SD)^f^	Failure^a^ (*n* = 307) mean (SD)^f^
Sociodemographic			
Age, in years	45.4 (±9.6)	43.7 (±9.2)	46.6 (±9.8)
CLBP history			
Duration of LBP, in years	12.5 (±10.8)	11.7 (±9.9)	13.0 (±11.3)
Primary outcome			
ODI oswestry disability index	41.4 (±14.1)	33.7 (±13.1)	46.8 (±12.0)
Secondary outcomes			
ZSDS Zung Self-rated Depression Scale	26.2 (±9.3)	24.4 (±9.9)	27.5 (±8.6)
NRS Numeric Rating Scale	60.7 (±21.1)	56.4 (±22.2)	63.7 (±19.8)
PCS Pain Catastrophizing Scale	22.9 (±8.9)	22.3 (±8.7)	23.4 (±8.9)
TSK Tampa Scale for Kinesiophobia	39.6 (±6.4)	39.0 (±6.5)	40.0 (±6.4)
PSEQ pain self-efficacy questionnaire	32.4 (±10.8)	36.3 (±10.1)	29.6 (±10.4)

^a^Success number of patients reaching at 1-year follow-up a ‘normal’ value of ten points on ODI (SD 12), Failure number of patients reaching at 1-year follow-up an ODI value of >22 points

^b^ *χ* ^2^ = 1.80, *p* = 0.21

^c^ *χ* ^2^ = 78.32, *p* < 0.001

^d^ *χ* ^2^ = 9.81, *p* < 0.05

^e^ *χ* ^2^ = 9.20, *p* < 0.05

^f^All variables *p* < 0.001

### Disability in the study sample

**Fig. 2:**
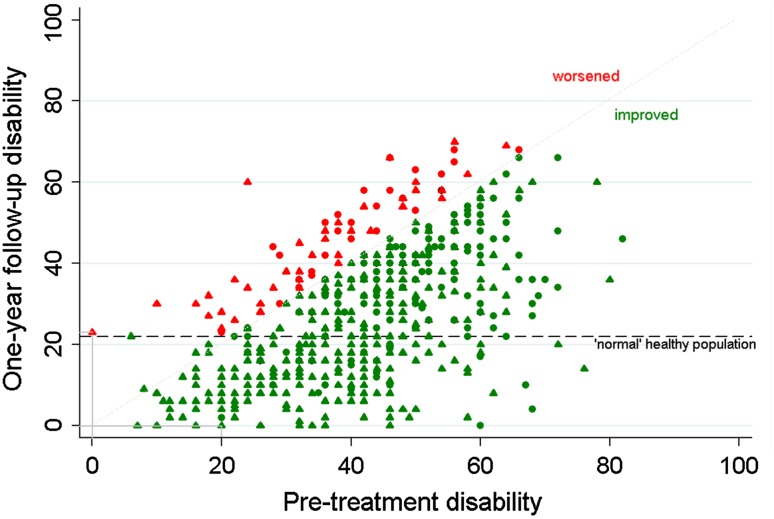
Functional status as measured with the ODI (0–100) in the study sample (*n* = 524). Oswestry disability index, with high values indicating high disability ∆ employed; ο unemployed at pre-treatment assessment; *red* *case*: pre-treatment ODI is 0, employed, and 1-year follow-up ODI is 24 (*worsened*); *green* *case*: pre-treatment ODI is 20, employed, and 1-year follow-up ODI is 0 (improved); *black* *dashed* *horizontal* *line* indicates the 22 point threshold for functional status [25] *Grey* *dotted* *diagonal* *line* is a reference line

As shown in Table 2, the mean pre-treatment disability (ODI) score for the study sample is comparable with the reported weighted mean score in chronic back pain populations [25] (41.4 [SD 14.1] and 43.3 [SD range 10.0–21.0], respectively). Figure 2 shows that most of the patients are improved at 1-year follow-up (green values). Moreover, at 1-year follow-up, 217 patients (41.4 %) reached the value for disability as measured in ‘normal’ populations; the green values below the black dashed horizontal line. Of these patients, 60 (27.7 %) already had an ODI pre-treatment value of 22 or less. At 1-year follow-up, the mean improvement in disability was 31.0 % in relation to the pre-treatment value (25th percentile 59.0 %; 50th percentile 32.3 %; 75th percentile 8.5 %).

**Table 2:** Means and standard deviations (SD) for the primary outcome of disability as measured with the oswestry disability index (ODI) in this study and in reference populations

	Mean	SD
RealHealthNL programme^a^ (*n* = 524)		
Pre-treatment assessment	41.4	14.1
One-year follow-up assessment	27.6	16.4
‘Normal’ population^b^ [25] (*n* = 461)	10.2	Range 2.2–12.0
Chronic back pain population^b^ [25] (*n* = 1,530)	43.3	Range 10.0–21.0

^a^Current study

^b^Values based on different study populations

### Prediction model for success at 1-year follow-up

Overall, small Pearson correlations were found between pre-treatment patient characteristics and pre-treatment values for primary and secondary outcome measures as well as the outcome of being successful on the ODI; Pearsons’ *r* ranging from <0.01 (pre-treatment pain duration) to 0.62 (pre-treatment disability). This means that no strong co-linearity exists between different variables and successful outcome. In Table 1, the pre-treatment characteristics are described for the success and failure groups after the programme. As shown in Table 1, all but one categorical variable (‘gender’; *χ* ^2^ = 1.80, *p* = 0.21) as well as all the continuous variables were significantly associated with the outcome of 1-year successful diminution of perceived disability. A univariate logistic regression model was built with all these variables entered in one block. Pre-treatment age categories, previous surgery, being employed as well as pre-treatment pain self-efficacy and pre-treatment disability appeared to be potential predictor variables. With a forward selection method and in one block, these variables were included in the model. The final prediction model revealed being employed (OR 3.61 [95 % CI 1.80–7.26]) and pre-treatment disability (OR 0.94 [95 % CI 0.92–0.97]) as significantly contributing factors for clinically relevant improvement in disability, defined as having values measured in ‘normal’ populations (Table 3). No interaction effects between different pre-treatment characteristics were found. Moreover, the results obtained by the ten databases that were generated from the MI-database produced the same result.

**Table 3:** Development of a multivariate logistic regression model for being successful at 1-year follow-up (*n* = 262; 50 % random selection of cases)

Forward selection method (final model)	Β (SE)	Wald	*p*	95 % confidence interval for exp (b)		
				Lower	Exp (b)	Upper
Included						
Step 1						
Functional status (ODI)	−0.07 (0.01)	37.35	0.00	0.91	0.93	0.95
Constant	2.39 (0.48)	24.97	0.00		10.88	
Step 2						
Employed	1.28 (0.36)	12.97	0.00	1.80	3.61	7.26
Functional status (ODI)	−0.06 (0.01)	24.32	0.00	0.92	0.94	0.97
Constant	1.62 (0.52)	9.69	0.002		5.05	
*R* ^2^ = 0.22 (Hosmer and Lemeshow), 0.17 (Cox and Snell), 0.23 (Nagelkerke)						

Model *χ* ^2^(2) = 62,136 *p* < 0.001; 66.8 % correct classification

*ODI* oswestry disability index

These results imply that a patient has a 1.3-fold risk of failure in the programme when not employed at the pre-treatment assessment. Moreover, the predictive value of disability is protective, meaning that for each point that the pre-treatment ODI score is closer to the normal value, the probability that the patient will meet the success criterion increases by 6.0 %. Overall, using this final model, 66.8 % of the participants had been correctly classified as being successful.

The validity of the model was checked with the remaining cases (*n* = 262; Table 4). A multivariate prediction model with the same variables as found in the prediction model that had been developed was built. As shown in Table 4, the results are comparable to those found using the other half of the dataset. However, most of the 95 % CI limits around the calculated OR’s (Exp [b]) are broader. This means that the model is less precise for the second half of the dataset, even though both the explained variance and the percentage participants correctly classified are higher than that found for the model developed with the first half of the dataset (*R* ^2^ = 40.0 % [Hosmer and Lemeshow]) and 75.0 %, respectively).

**Table 4:** Validation of developed multivariate logistic regression model for being successful at 1-year follow-up (*n* = 262; 50 % remaining cases)

Forward selection method (final model)	Β (SE)	Wald	*p*	95 % confidence interval for exp (b)		
				Lower	Exp (b)	Upper
Included						
Step 1						
Functional status (ODI)	−0.09 (0.01)	50.74	0.00	0.88	0.91	0.93
Constant	3.70 (0.56)	43.20	0.00		40.35	
Step 2						
Employed	1.84 (0.40)	21.39	0.00	1.96	6.29	13.70
Functional status (ODI)	−0.09 (0.01)	36.47	0.00	0.89	0.92	0.94
Constant	2.80 (0.61)	21.23	0.00		16.38	
*R* ^2^ = 0.40 (Hosmer and Lemeshow), 0.25 (Cox and Snell), 0.34 (Nagelkerke)						

Model *χ* ^2^(2) = 101,651, *p* < 0.001; 75.0 % correct classification

*ODI* oswestry disability index

To predict a patient’s probability of being successful after having participated in the programme, the identified contributing factors were included in the logistic function. For example, a patient who is employed at pre-treatment assessment, and who has a pre-treatment ODI value of 35, is classified as probably being successful in the programme (*p* = 0.70). On the other hand, a patient who is not employed at pre-treatment assessment, and who has a pre-treatment ODI value of 60, will probably be a failure in the programme (*p* = 0.13).

### Contribution of psychological distress

As psychological distress appeared not to be a predictive factor for treatment outcome, a separate analysis was not performed. Therefore, there is no evidence to support the hypothesis that an association exists between ‘depressed mood’ and failure 1 year after the programme.

## Discussion

The most important finding of this longitudinal study is that being employed and the level of disability before treatment are predictive factors for relevant improvement in CLBP patients’ functional status at 1-year follow-up. In contrast to our expectation, the pre-treatment degree of experienced pain intensity and belief in one’s ability to manage and to cope with CLBP complaints appeared not to be predictive of outcome. Moreover, the results revealed that 1 year after the programme, highly distressed patients who were referred to the programme were not at risk of being a failure.

Previously, this CPP programme has been evaluated for patients who met the inclusion criteria [20, 21]. The present analysis was conducted to determine whether it would be possible to enhance the efficacy of the programme by further patient selection by identifying a subgroup of patients who could benefit of the programme. As the main goal of the intervention is to improve disability, success at 1-year follow-up was defined as having reached 22 points or lower on the ODI. We reasoned that less change is not clinically relevant.

A minimal clinical important difference (MCID) of ten points on the ODI has been recommended as a measure for clinical relevancy in CLBP [33]. Although consensus has been reached for this MCID value, the value is still arbitrary because some of the studies upon which the consensus is based contain heterogeneous CLBP population samples and were derived from primary care [33]. It is difficult to measure what is clinically relevant to patients [34]. Patients who are highly disabled at pre-treatment assessment and who did reach the MCID value after treatment could be classified as improved success whilst in fact they are still disabled. Therefore, we decided to use ODI values seen in ‘normal’ healthy populations as a measure of success. The current study results show that at 1-year follow-up 217 patients (41.4 %) reached this ODI value. With the exception of one study, which was performed in primary care [35] and included CLBP patients who were still at work and who were less disabled (ODI 20 [range 2–52]), we are not aware of any studies performed in secondary or tertiary care investigating factors predicting a functional outcome related to ‘normal’ and healthy populations.

### Prediction model: pre-treatment ‘employed’ and pre-treatment ‘disability’

Being employed appeared to be the most important predictive factor (OR 3.61 [95 % CI 1.80–7.26]; dichotomised). To a lesser extent, the level of pre-treatment disability predicts the outcome (OR 0.94 [95 % CI 0.92–0.97]; decrease per point on ODI). These findings are consistent with the results of the systematic review by van der Hulst et al. [6]. We recommend screening CLBP patients for these factors. It is known that CLBP patients who are significantly disabled and who are absent from work pre-treatment have a poor outcome [36, 37]. The ODI might have screening potential as it has been shown to be of predictive value for chronicity [37]. Patients who are moderately disabled and who are at least partially employed before treatment could be given a higher priority for entry into a CPP programme. From an organisational and economic perspective, patients who are at work and who are mildly disabled might benefit from a shortened programme. To substantiate these ideas, more research is needed.

In the current study, the prediction model (Table 3) has wider confidence intervals for the validation model (Table 4), and a lower explained variance (*R* ^2^ 22 % versus 40 % [Hosmer and Lemeshow]), resulting in a greater number of cases correctly classified (67 versus 75 %) for the validation model. Because of these discrepancies and to estimate the stability of the prediction model, we performed a post hoc multivariate logistic regression analysis on the random sample (*n* = 252) using a bootstrap procedure that is 500 repeated samples with replacement. All potential prediction variables were then entered in one block. This result is comparable to the final prediction model (Model *χ* ^2^ [5] = 68,157 *p* < 0.001; *R* ^2^ 24 % [Hosmer and Lemeshow]; 23 % [Cox and Snell]; 31 % [Nagelkerke]; 70 % correct classified). Based on these results, we conclude that the final prediction model, as initially developed, is robust. This model explains 22 % (Hosmer and Lemeshow) of the total variance. Moreover, 67 % of the patients were correctly classified. Although inconsistent evidence does exist for predictive factors that were identified for outcome of interventions with a physical and cognitive behavioural approach, a comparable and typical low amount of explained variance has been found [38–41]; as well as the percentage correctly classified patients [42]. Because physical and psychosocial factors only marginally contribute to treatment success, other non-specific or moderating factors such as clear treatment rationale, a highly structured programme, providing a pressure-cooker model programme, the dose of treatment, skilful staff, and the patient’s readiness to change pain-related behaviour have been proposed as being predictive for a successful outcome [11, 43, 44]. There are two increasingly suggested specific contributing factors to functional treatment outcome in chronic musculoskeletal pain: expectancy of treatment outcome [45] and central sensitisation [46–48]. Central sensitisation includes features of referred pain, hypersensitivity to peripheral stimuli and neuropathic pain which are felt to represent peripheral manifestations of augmented central pain sensations. However, further research is required to determine which specific factors contribute to a successful outcome for CLBP patients in a CPP programme.

Some inconsistent qualitative evidence has been reported which is related to other potential and a priori predictive factors that might be expected for this study: experienced pain intensity [6, 49], gender [7, 23], or self-efficacy [35, 50, 51]. However, no support for these predictive factors could be found in the present study. It has also been suggested that improvement of dysfunctional cognitive behavioural factors such as catastrophizing cognitions and fear of movement behaviour might contribute to a successful outcome [11, 52]. This suggestion is endorsed by the fear avoidance model which postulates a causal relationship between pain catastrophizing, fear of movement, disability and experienced pain severity [4]. Some studies have concluded that the impact of these dysfunctional cognitive behavioural factors on outcome measures as pain as well as functional status is diminished [15, 53] or is even absent [6], which is consistent with the results of the present study.

Studies investigating the predictive value of psychological distress have only yielded inconclusive and tentative evidence [6, 15]. Self-rated depressive mood has been reported to be of prognostic value [8, 18, 22, 39, 54]; furthermore, it has been suggested that patients with reported symptoms would benefit less from a multidisciplinary programme compared to patients with no or only mildly depressive symptoms [7, 18, 23]. In the current study, despite a small association between the level of distress and being successful at 1-year follow-up (Pearson’s *r* −0.23, *p* < 0.001), no predictive value of psychological distress could be found in the final prediction model. This means that CLBP patients who are distressed at pre-treatment assessment might benefit from a CPP programme.

### Strengths and limitations

The strengths of this study are the large sample size (*n* = 524) and the wide range of available pre-treatment data. This means that there was enough statistical power to study the contribution of the different potential predictive factors towards successful treatment outcome over time. Although data were missing on at least one assessment for 67 (13 %) patients, no pre-treatment differences between non-responders and responders were seen. Our main results are based on the MI technique. MI is a technique that depends on model-based imputation of multiple values for each missing observation instead of only one estimate as in single imputation techniques. The major advantage of this method, over single imputation techniques or ‘complete cases only’, is that it does not underestimate variability. Single imputation methods could result in the estimated standard errors being too small, whereas multiple imputation results in the correct magnitude for estimated standard errors and confidence intervals [55, 56], i.e. these imputed values reflect the uncertainty in estimation caused by the missing values [56]. Thus, the information contained within the missing data seems similar in nature to the information actually documented. This implies that the conclusions based on the results obtained with MI are robust. Moreover, the large study sample gave us the opportunity to develop a prediction model in a 50 % random sample of the original set and to validate and check this final model with the remaining data.

Limitations in this study include possible selection bias. Therefore, generalisation to common clinical practice is limited as our findings are theoretically relevant only to specialised back care. There are no data for those patients not selected (28 %), it is possible that other factors could be predictive for a successful treatment outcome. It is possible that these patients were not ready or motivated to change pain-related behaviour. Although a selection criterion for treatment, we neither assessed this factor in a valid and reproducible way at pre-treatment nor assessed it systematically over time. Further research is needed to assess this factor and to evaluate its contribution to the outcome.

## Conclusion

The study results imply that CLBP patients who are in work and mild to moderately disabled at the start of a CPP programme benefit from it and have a successful treatment outcome. In these patients the disability falls to values seen in healthy populations. Even psychologically highly distressed patients may respond positively to this programme. The limited number of predictive indicators is extremely useful. The small set of easily identified indicators might speed up assigning priority for programme entry and triage to alternative treatment regimes.
